# Executive functions predict time reference processing in French-speaking people with Alzheimer’s disease

**DOI:** 10.1038/s41598-025-20297-y

**Published:** 2025-10-17

**Authors:** Evodie Schaffner, Mélanie Sandoz, Noémie Auclair-Ouellet, Jean-François Démonet, Marion Fossard

**Affiliations:** 1 Institute of Logopedic Sciences, Faculty of Letters and Humanities, University of Neuchâtel, Rue de la Pierre-à-Mazel 7, 2000 Neuchâtel, Switzerland; 2 School of Communication Sciences and Disorders, McGill University, 2001 av. McGill College, Montreal, QC H3A 1G1 Canada; 3 Leenaards Memory Center, Lausanne University Hospital, Mont-Paisible 16, 1011 Lausanne, Switzerland

**Keywords:** Alzheimer disease, Time reference, Tense, Aspect, Executive functions, Working memory, Cognitive ageing, Alzheimer's disease

## Abstract

The ability to express time through language, known as time reference, is impaired in people with Alzheimer’s disease. While cognitive impairments have been documented in this population, particularly in executive functions, few studies have examined how these deficits impact time reference abilities, including tense and grammatical aspect. Since producing time reference requires the integration of grammatical, conceptual, and subjective information, potentially demanding in processing resources, the aim of this study was to investigate whether the cognitive profile (i.e., executive function abilities) of French-speaking people with biologically probable Alzheimer’s disease determines their ability in time reference. Verb inflection tasks and cognitive tests were administered to 21 people with a diagnosis of Alzheimer’s disease confirmed by cerebrospinal fluid or amyloid positron emission tomography (PET) biomarkers and a control group. Results revealed that individuals with Alzheimer’s disease have difficulty with tense and aspect marking, with verbal working memory, inhibition, and mental flexibility playing a significant role in time reference processing. These findings suggest that deficits in executive functions impact the ability of French speakers with Alzheimer’s disease to mark tense and grammatical aspect, highlighting the cognitive basis of time reference impairments in this population.

## Introduction

Time is a fundamental aspect of the human experience. Each event or personal experience is anchored in a specific temporal framework and consists of actions occurring within defined time boundaries. As human beings, we can perceive time, navigate mentally through it, conceptualize it, and express it. Expressing time through language enables us to share our personal experiences, participating in the construction of our social lives and providing us with a sense of self^1,2^. When sharing a personal experience, two main elements of information are needed, i.e., when and how did this experience occur. Time reference specifies when an experience take place and thus corresponds to the linguistic expression of time. It is then at the interface between the concepts of time and language. It gives information about the localization and the progress of the reported action and therefore constitutes an important part of efficient communication. Time reference can be impaired in acquired language disorders such as fluent or non-fluent aphasia^3–9^, semantic variant primary progressive aphasia^10,11^, or Alzheimer’s disease (AD)^12–15^. Interestingly, although the impairment manifests similarly across these disorders - specifically, difficulties in verb inflection - the underlying mechanisms may differ and remain poorly understood, particularly in individuals with AD. While AD is primarily characterized by episodic memory deficits^16^, executive dysfunction is also a core feature of this disease^17,18^. Changes in executive functions such as working memory have been shown to affect language in people with AD^19–21^. However, their effect on the production of time reference, which requires the integration of several sources of information, is not well understood. This raises the question of whether executive dysfunction might influence how time is expressed through language.

Time reference allows the expression of time in language and specifically, of the concepts of past, present, and future as well as the degree of completion of an event. Because every human experience is situated within these temporal dimensions, processing time reference is a cognitively complex process. Speakers must indeed first consider the temporal status of an event – whether it is past, present or future, and completed or ongoing – and then transcribed it in language. In tense languages such as French or English, this process relies on two primary grammatical categories: tense and grammatical aspect^22–24^, which contribute to event localization in time (past, present or future) and provide information about its internal structure (completed or ongoing). While some authors distinguish grammatical aspect from time reference^13,25,26^, others consider it as an integral part, like tense. This is particularly evident in languages such as French, where tense and aspect are inextricably embedded in certain verbal forms^22–24^. In this perspective, aspect not only reflects the internal progression of an action but also helps situate it on the temporal axis by indicating its course over time^22–24^. For example, in the sentence “Hier, je marchais quand j’ai rencontré Pierre” (“Yesterday, I was walking when I met Pierre”), the inflected verb forms “je marchais” (imperfective form: “I was walking”) and “j’ai rencontré” (perfective form: “I met”) not only establish the temporal framework of the past event but also indicate its progression over time. The first verb form indicates that the speaker was walking when Pierre arrived. This action was an ongoing process, began in the past but was not yet finished. Conversely, the action of “meeting Pierre” was brief, with a clearly defined beginning and end. In French, tense and aspect are expressed through verbal inflection and temporal adverbs^23^. Tense establishes a temporal link between an event and a temporal anchoring point^27^. It determines whether an event precedes, coincides with, or follows the speech act, corresponding to past, present, and future, respectively^28^. In French, tense is marked through inflectional morphemes on verbs (e.g., je march***e*** vs. je march***ais***; I walk vs. I was walking) as well as temporal adverbs (e.g., hier, aujourd’hui, demain; yesterday, today, tomorrow). While tense indicates *when* an event occurs, grammatical aspect conveys *how* it unfolds, by expressing its degree of completion^22–24^. A perfective aspect presents an event as a bounded whole, while an imperfective aspect presents it as ongoing. In French, the perfective-imperfective opposition is essentially valid in past tenses, with the “indicatif passé composé” or “indicatif passé simple” (perfectives) typically opposed to the “indicatif imparfait” (imperfective). As with tense, grammatical morphemes (e.g., j’ai cour***u*** / je cour***us*** vs. je cour***ais***; I was running vs. I ran; perfective vs. imperfective) and adverbs (e.g., mardi dernier vs. chaque mardi; last Tuesday vs. every Tuesday; perfective vs. imperfective) contribute to marking grammatical aspect. Additionally, aspect conveys the speaker’s subjective perspective on an event^22,24,27^. Indeed, the speaker is free to indicate their perception of the event, either as a complete whole (comprising its end, which is expressed with the perfective aspect) or as an ongoing process, viewed from an internal perspective (without the end, which is expressed with the imperfective aspect).

Research on time reference in people with AD is relatively recent, as morphosyntactic abilities were long thought to be preserved in this population^29^, and thus were not systematically assessed. To our knowledge, only a few studies using connected speech (e.g., picture description, semi structured interview) have shown that AD progression leads to fewer inflected verbs^30^ and increased inflection errors^31^. Few of them have however specifically examined time reference through specific verb inflection tasks, like sentence completion tasks, controlling for variables known to be involved in verb inflection (e.g., verb regularity, tense, agreement)^4,7,8,32,33^. Focusing mainly on Greek, a language in which tense and aspect greatly interact, these studies showed that tense and aspect are impaired in people with AD^12–15^. Much of what is known about time reference production then stems from aphasia studies^4,7,8,26,32–34^, with recent work exploring cognitive predictors of tense production^6,9,35^. Building on theories suggesting that processing resources influence time reference production (see for instance^4,36,37^), authors working in aphasiology demonstrated that working memory capacity predicts tense production not only in individuals with aphasia but also in healthy aging^6,9,35^. This effect has recently been extended to AD, where working memory has similarly been found to predict tense production^12^. Yet, to date, no other studies have explored this relationship in depth within the AD population. Given that individuals with AD, like those with aphasia, often have difficulty with processing resources^19,20^, it is quite plausible that theoretical frameworks developed in aphasiology may also be applicable to AD.

For instance, the PAst DIscourse LInking Hypothesis (PADILIH)^4^ poses that the reference to past events is more difficult to process compared to the reference to present or future events due to the non-alignment of the moment of the event (past) and that of the time of speech (present). This non-alignment then requires linking the sentence to discourse and would be more demanding in terms of processing resources^38,39^. According to the PADILIH, the future tense is considered a special case of the present. Since the event being reported has not yet happened, it cannot be referred to directly. This hypothesis has mainly been tested in aphasia and is based on the idea of a necessary linguistic link between sentence and discourse. Given the specific cognitive profile of AD, and in particular the general cognitive decline already found at a prodromal stage^17,18^, it could be asked whether executive skills also intervene in the construction of this link in AD.

Another perspective on the role of processing resources in time reference production comes from findings showing that tense and aspect are more impaired than agreement in individuals with aphasia^35–37^, and with AD^12–15^. A possible explanation lies in the varying cognitive demands required for producing agreement, tense, and aspect. Agreement is thought to require fewer processing resources since it is a local phenomenon involving only syntactic grammatical processing^36,37^. In contrast, tense is more demanding because it requires integrating conceptual information alongside grammatical information. Multiple temporal references must be processed simultaneously, including the event itself, the time of speech, and their relationship. This process follows objective criteria, such as determining whether an event occurs before, during, or after the speech moment, classifying it as past, present, or future^37^. Grammatical aspect may be even more demanding, as it involves processing additional abstract concepts such as habituality, continuity, and perfectivity^22,37^. Beyond information linked to agreement or tense, aspect requires considering the speaker’s intentions and subjective perspective—specifically, whether they present the action as completed (perfective) or ongoing (imperfective). Aspect would then be particularly demanding in processing resources.

Further research is now needed to better understand how time reference is impaired in AD and more specifically, to clarify what is behind the notion of “processing resources”. While some studies have compared the production of agreement, tense, and aspect - suggesting that cognitive load varies depending on the concept being expressed - few have directly examined the role of executive functions in time reference production. In addition, although working memory has been shown to impact time reference in individuals with AD^12^, further investigation is needed to fully understand its contribution to time reference processing. Moreover, working memory may not be the only cognitive function involved. Other abilities, such as mental flexibility or inhibition, have been linked to difficulties in perceiving or expressing time in some neurodegenerative diseases^40–43^. Inhibition and flexibility could then be particularly involved when time or perspective shifts are required. The cognitive demand could also depend on the nature of the task. Therefore, the role of these additional cognitive functions in time reference processing warrants further exploration.

This study aims then to examine how the cognitive profile of French-speaking individuals with AD affects their ability to produce time reference at the levels of tense and aspect. Research on time reference in AD remains limited and has primarily focused on Greek, a language where tense and aspect greatly interact^26^. French is a morphologically complex language, where tense and aspect interact systematically only in past tenses, thought to be particularly challenging to produce^4^. Studying time reference in French will provide valuable insights into the cognitive processes involved in this process.

Furthermore, rather than exploring whether time reference is impaired in people with AD and comparing tense and aspect production, this study aims, using predictive analyses, to assess how executive functions predict time reference production in individuals with AD. Indeed, since French marks grammatical aspect only in the past tense, the comparison of tense and aspect production in this language differs from that in languages where the two interact differently. While working memory has been shown to influence time reference^12,35^, other executive functions, such as mental flexibility and inhibition, may also contribute to verbal inflection. We therefore hypothesize that working memory, mental flexibility, and inhibition will predict time reference abilities in individuals with AD. Additionally, we anticipate that the past tense will be more impaired than the present and future tenses, due to the greater cognitive demands involved in processing an event that is temporally distant from the moment of speech^4^. In contrast, the present tense aligns with the time of utterance, requiring less executive control. Regarding grammatical aspect, we expect the imperfective aspect to be more impaired than the perfective aspect, as it requires shifting perspective within an event, making it cognitively more demanding. The perfective aspect, by contrast, presents an action as completed and viewed from a present perspective^22,24,27^.

## Methods

### Participants

Forty-two French-speaking participants, divided into two groups, took part in the study. Twenty-one participants with a diagnosis of AD confirmed by cerebrospinal fluid or amyloid PET biomarkers were included in the AD group, and twenty-one healthy participants matched to the AD group in age, gender and education were included in the control group (see Table 3). An a priori power analysis was conducted to determine the sample size. Results showed that, with a statistical power of 0.85, a sample size of 21 participants in both groups is sufficient to detect significant differences between the groups at the alpha level of 0.05.

For both groups, the inclusion criteria were (1) being a native French speaker or have a very good command of French (i.e., having completed compulsory school and training in French) and (2) having normal or corrected vision and hearing. The exclusion criteria were having (1) a history of psychiatric disorders as described by the DSM-5^44,45^, (2) symptoms of an alcohol or drug dependence disorder according to the DSM-5 criteria, (3) focal intracerebral lesions of a post-traumatic or cerebrovascular nature.

Participants of the AD group were recruited from the Leenaards Memory Center of the Lausanne University Hospital in the French-speaking part of Switzerland. In addition to the selection criteria presented above, the participants in this group had to (1) have been diagnosed with probable AD according to the revised NINCDS-ADRDA criteria^16^, (2) have a global Clinical Dementia Rating (CDR) score of 0.5 to 1, (3) have mild to moderate cognitive impairment according to the Montreal Cognitive Assessment (MoCA) score^46^, and (4) show biomarkers linked to the AD diagnostic according to the ATN (amyloid, tau, neurodegeneration) classification^47^.

Participants in the control group had to show preserved global cognitive functioning by obtaining a score equal to, or greater than, 26/30 on the MoCA^46^ and a score equal to or greater than the threshold scores stratified for age and educational level on the Dépistage des Troubles du langage chez l’Adulte et la personne âgée (DTLA) test which is a screening test of language, used in French-speaking countries^48,49^.

This study was approved by the local ethics committee (Commission cantonale d’éthique de la recherche sur l’être humain du Canton de Vaud – CER-VD) on 9 March 2021 (approval no. 2020–02723) and performed in accordance with the Declaration of Helsinki. To take part in the study, all participants were required to give their written consent after having been informed orally and in writing of the objectives of the study.

#### Material and procedure

Participants were involved in a larger research project on time reference (tense and grammatical aspect) in AD^50^ and vascular aphasia^5,6^ in French and underwent a cognitive abilities and language assessment before performing the two tasks assessing tense and the two tasks assessing grammatical aspect. To evaluate tense and grammatical aspect in French, four sentence completion tasks that specifically target time reference were built. These tasks incorporated temporal and aspectual cues unique to French - such as characteristic adverbial expressions - to ensure the assessment accurately target tense or grammatical aspect.

All the tasks were completed within three sessions of one and a half to two hours for the AD group and two sessions of two hours for the control group. Breaks were provided to avoid fatigue effects. All participants were tested individually in a quiet room, at the Leenaards Memory Center or in their homes for the AD group, and at the University of Neuchâtel or in their homes for the control group.

#### Tasks assessing tense

**Fig. 1:**
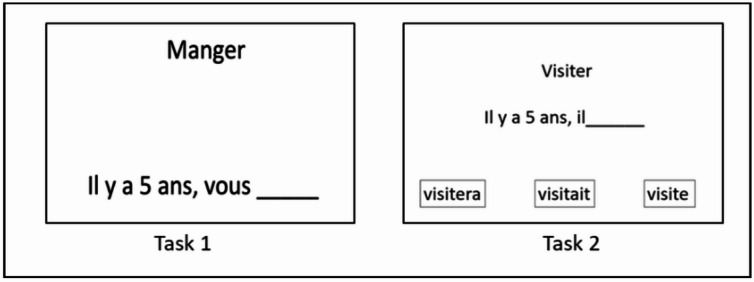
Illustration of experimental tasks for assessing tense. Task 1: Eat → 5 years ago, you_____; Task 2: Visit → 5 years ago, he_____ will visit visited visits.

Two sentence completion tasks were used to assess tense marking (see Fig. 1 below), namely one task of Production of an inflected verb form (Task 1) and one task of Selection of an inflected verb form (Task 2). They were built to assess both the encoding and the retrieval components of verb inflection^9^. Indeed, in Task 1 participants had to encode and select the correct temporal information of the inflected verb form to then retrieve and produce the appropriate inflected verb form. It aims then at assessing how participants can produce inflected verb forms concatenating the verbal stem with the inflection corresponding to the temporal context of the sentence provided by a temporal adverbial as temporal cue. In Task 2, the encoding was not necessary, but participants were required to retrieve the correct inflected verb form. This task aims to assess how participants are able to select inflected verb forms identifying the correct diacritical features of tense (i.e., past, present or future) in an inflected verb form corresponding to the temporal context of the sentence provided by a temporal adverbial as temporal cue. Despite encoding and retrieval, both tasks may tax processing resources as different kinds of information (e.g., personal pronoun, time, etc.) were provided and had to be addressed.

In Task 1, the participant had to complete an incomplete sentence by producing an inflected verb form at the past, present or future tense depending on temporal cues given by a temporal adverbial (e.g., Past “Manger : Il y a 5 ans, vous_____” expected answer = mangiez → “Eat: Five years ago, you_____” expected answer = ate; Present “Réfléchir : Maintenant, tu_____” expected answer = réfléchis → “Think: Now, you_____” expected answer = think; Future “Fuir : Dans plusieurs années, vous_____” expected answer = fuirez → “Flee: In several years, you_____” expected answer = will flee).

In Task 2, the participant had to complete an incomplete sentence by selecting an inflected verb form among three possible given answers, corresponding to the past, present or future tense, depending on temporal cues given by a temporal adverbial (e.g., Past “Manger : Il y a cinq ans, il_____ mange mangera mangeait” expected answer = mangeait → “Eat: Five years ago, he_____ eats will eat ate” expected answer = ate; Present “Réfléchir: Maintenant, il_____ réfléchira réfléchit réfléchissait” expected answer = réfléchit → “Think: Now, he_____ will think thinks thought” expected answer = thinks; Future “Fuir : Dans plusieurs années, il_____ fuira fuyait fuit” expected answer = fuira → “Flee: In several years, he_____ will flee fled flees” expected answer = will flee).

Both tasks were computerized and presented in a PowerPoint document: an infinitive verb form appeared on the screen, followed by the sentence to complete. The experimenter instructed the participant this way for Task 1: “You will see and hear a verb followed by a short sentence to complete. You must complete the sentence out loud using the verb given. We will start with 3 practice sentences during which I will show you how to do the exercise.” For Task 2, three possible answers followed the presentation of the incomplete sentence and the instruction was: “You will see and hear a verb followed by a short sentence to complete. You must complete the sentence by pointing to the answer among the three options with your finger on the screen. We will start with 3 practice sentences during which I will show you how to do the exercise.” Then, the experimenter read the item as it appeared on the screen to give orally and written information to the participant. Each task contained three training items (one item for each condition of tense) in which the experimenter demonstrated how to perform the task. These training items were followed by 48 experimental items and the order of the presentation was pseudorandomized.

All of these items were controlled for frequency using the Lexique database^51^, for the length of the infinitive form (1–4 syllables), and for lexical aspect (activities only). In Task 2, agreement was also controlled with the sentence given in the third person masculine singular (il). Table 1 below shows the mean and standard deviation for the frequency and the length of the items.

**Table 1:** Mean and standard deviation of frequency and length (number of syllables) for items included in tasks assessing tense.

Task		Oral frequency		Written frequency		Length	
		Mean	SD	Mean	SD	Mean	SD
Task 1	VR	*40.22*	*18.86*	*45.74*	*20.31*	2.75	0.45
	VPR	*43.35*	*28.67*	*42.80*	*26.02*	2.50	0.52
	VIRSC	43.21	20.82	62.40	35.36	2.25	0.87
	VIRAC	32.41	22.44	42.33	19.74	1.83	0.58
	Present	44.98	27.61	48.80	27.27	2.31	0.60
	Past	33.81	14.46	44.00	21.49	2.19	0.66
	Future	40.60	23.92	52.16	31.27	2.50	0.82
	2nd p.s.	38.47	24.67	49.74	31.07	2.63	0.65
	2nd p.p.	41.12	20.98	46.89	21.87	2.04	0.62
Task 2	VR	43.57	28.40	46.18	24.48	2.79	0.43
	VPR	39.30	16.40	41.59	21.40	2.40	0.52
	VIRSC	43.21	20.82	62.40	35.36	1.83	0.58
	VIRAC	32.41	22.44	42.33	19.74	2.33	0.89
	Present	44.98	27.61	48.80	27.27	2.38	0.62
	Past	33.81	14.56	44.00	21.49	2.19	0.66
	Future	40.60	23.90	52.16	31.27	2.50	0.83

SD = standard deviation; VR = regular verb; VPR = pseudo-regular verb; VIRSC = irregular verb without stem change; VIRAC = irregular verb with stem change; 2nd p.s. = second person singular (tu); 2nd p.p. = second person plural (vous).

In both tasks, tense (past, present, future) and regularity (regular verbs, non-regular verbs) were manipulated. In Task 1, agreement was also manipulated with the sentence given either in the second person singular (tu) or in the second person plural (vous). This choice was made to avoid automatism or perseverance, to complicate the task, and because of the clear phonological differentiation of the inflected verb forms associated with both pronouns (e.g., tu manges [mãӡ] vs. vous mangez [mãӡe]).

For each item, 1 point was given for the expected answer or an autocorrection, and 0 points for an incorrect answer, a non-response or a delayed response (given more than 10 s after the presentation of the item). No feedback was given to the participant during the experimental items.

For both tasks, three times as many stimuli had been pilot-tested with 50 healthy French-speaking people. This was done in order to retain only the most discriminating stimuli (48 stimuli in total for each of the two tasks) and ensure that participants understood the differences between the tasks and that the temporal cues were strong enough to preferentially lead to the expected response. Analyses were performed to test the validity of the final tasks with people with fluent and nonfluent aphasia showing good convergent, divergent and construct validities^52^.

#### Tasks assessing grammatical aspect

**Fig. 2:**
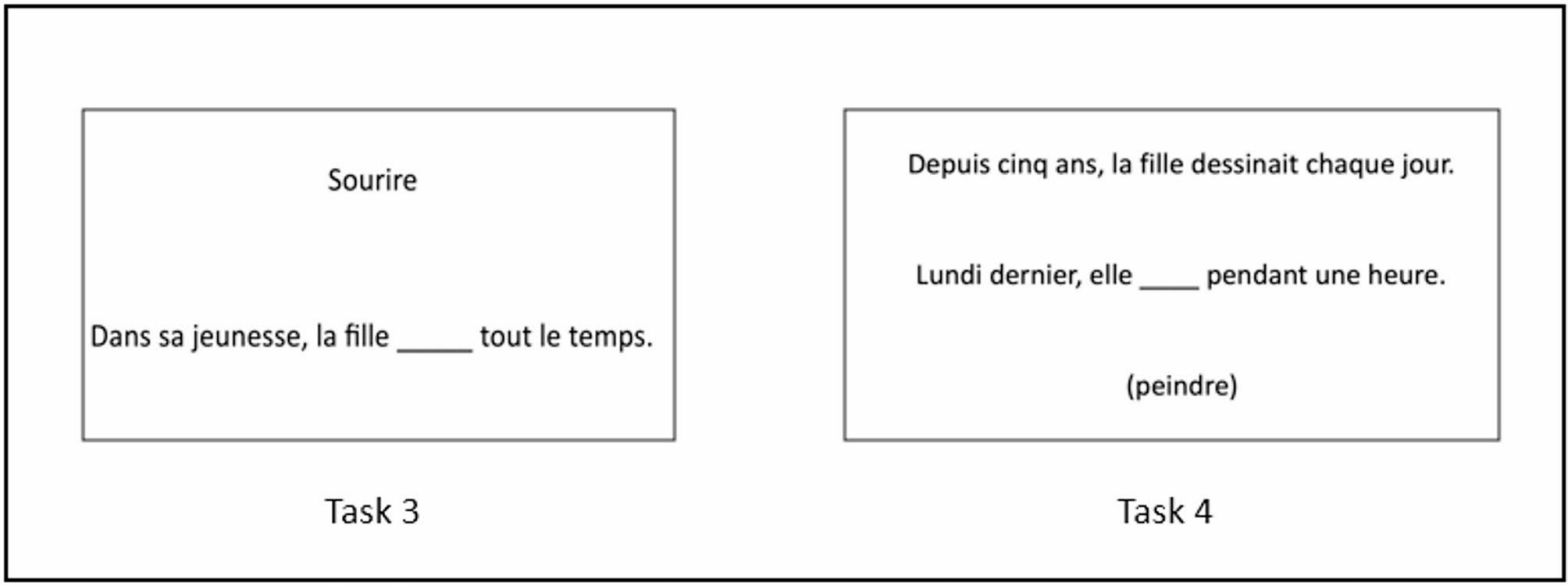
Illustration of experimental tasks for assessing grammatical aspect. Task 3: Smile → In her youth the girl _____ always; Task 4: Paint → For 5 years, the girl had been drawing every day. Last Monday, she ____ for an hour.

Two tasks of production of an inflected verb form at past tense (tasks 3 and 4) were used to assess the production of grammatical aspect through verbal inflection capacity (see Fig. 2 below). As for tasks assessing tense, both tasks assessing grammatical aspect focused on the encoding and the retrieval of the inflected verb forms. Tasks 3 and 4 targeted the production of an inflected verb form and both tasks may possibly tax processing resources as several information such as personal pronouns and aspect need to be considered. Moreover, Task 4 was built to focus on aspectual update and constrain the participant to change and update their aspectual perspective on the past event depicted in the sentence to complete. This task could be particularly demanding as the participant has to shift from a past event to another, taking a new aspectual perspective.

In Task 3, the participant had to complete an incomplete sentence by producing a past inflected verb form in a simple context, depending on temporal and aspectual cues given by some adverbials (e.g., Imperfective “Sourire : Dans sa jeunesse, la fille_____tout le temps” expected answer = souriait → “Smile: In her youth, the girl_____always” expected answer = was smiling; Perfective “En cinq ans, le garçon_____une fois” expected answer = a souri → “Smile: In five years the boy_____once” expected answer = has smiled).

In Task 4, the participant had to complete an incomplete sentence by producing a past inflected verb form depending on temporal and aspectual cues given by some adverbials in a context demanding aspectual update (e.g., Imperfective “Peindre : Le 6 juillet 2014, la femme a dessiné jusqu’à midi. A cette époque, elle_____chaque après-midi” expected answer = peignait → “Paint: On July 6, 2014, the woman drew until noon. At that time, she_____every afternoon” expected answer = was painting; Perfective “Peindre : Depuis cinq ans, la fille dessinait chaque jour. Lundi dernier, elle_____pendant une heure” expected answer = a peint → “The girl has been drawing every day for five years. Last Monday, she_____for an hour” expected answer = painted).

Both tasks were also computerized and presented in a PowerPoint document: Task 3 followed the same principle as Task 1 except that participant had to produce a past inflected verb form. An infinitive verb form appeared on the screen, followed by the sentence the participant had to complete orally with a past inflected verb form. The experimenter instructed the participant this way: “You will see and hear a verb followed by a short sentence to complete that presents an ongoing or completed action. You must complete the sentence out loud by inflecting the verb in the past tense. We will start with 4 practice sentences during which I will show you how to do the exercise.” For Task 4, which focused on aspectual update, a completed sentence depicting a past event appeared on the screen, followed by an incomplete sentence with an infinitive form verb in brackets. The instruction was: “You will see and hear a first sentence. Then, you will see and hear a second sentence that presents an ongoing or completed action and a given verb in parentheses. You must complete this second sentence out loud by inflecting the given verb in the past. Only verbs inflected in the past are allowed. We will start with 4 practice sentences for which I will show you how to do the exercise.” Since the aspectual form given in the first sentence was different from the one to be produced, this task more specifically targeted aspectual updating. Indeed, aspectual cues in the sentence to complete were chosen so that participants had to change their aspectual perspective and produce the reverse grammatical aspect from the first sentence (e.g., perfective form in the first sentence but imperfective form to produce in the second sentence and inversely). Fillers with the same aspectual form in both sentences were then included in this task to avoid automatisms. The eight fillers were not considered in the total score.

For both tasks, the experimenter read the item as they appeared on the screen giving oral and written information to the participant. The experimenter demonstrated how to perform each task presenting four training items. These training items were followed by 24 experimental items (12 verbs presented in both conditions). Two lists were created for the experimental items to ensure that each verb was presented in only one condition per list. The two lists were assessed separately, in different sessions. All of these items were controlled for frequency using the Lexique database^51^, for the length of the infinitive form (1–4 syllables), for regularity (no regular verbs), tense (past tense), agreement (third person of the singular) and for lexical aspect (activities). Aspect was manipulated (12 verbs for imperfective form and 12 verbs for perfective form). Table 2 below shows the mean and standard deviation for the frequency and the length of the items.

**Table 2:** Mean and standard deviation of frequency and length (number of syllables) for items included in tasks assessing aspect.

Task		Oral frequency		Written frequency		Length	
		Mean	SD	Mean	SD	Mean	SD
Task 3	*VPR*	26.60	24.45	23.45	15.34	2.00	0.0
	VIRSC	30.70	19.68	47.31	28.97	1.25	0.46
	VIRAC	31.96	20.80	90.97	106.23	1.75	0.46
	Perfective	29.75	21.37	53.91	69.17	1.67	0.49
	Imperfective	29.75	21.37	53.91	69.17	1.67	0.49
Task 4	*VPR*	26.60	24.45	23.45	15.34	2.00	0.0
	VIRSC	30.70	19.68	47.31	28.97	1.25	0.46
	VIRAC	27.95	16.35	39.74	12.26	1.50	0.53
	Perfective	28.83	20.46	36.84	22.32	1.58	0.51
	Imperfective	28.83	20.46	36.84	22.32	1.58	0.51

SD = standard deviation; VPR = pseudo-regular verb; VIRSC = irregular verb without stem change; VIRAC = irregular verb with stem change.

For each item, 1 point was given for the expected answer or an autocorrection, and 0 points for an incorrect answer, a nonresponse or a delayed response (given more than 10 seconds after the presentation of the item). No feedback was given to the participant during the experimental items. As for tasks assessing tense, both tasks assessing grammatical aspect were pilot tested with healthy people with three times as many stimuli to retain only the most discriminating stimuli (24 stimuli in total for each of the two tasks) and ensure that participants understood the differences between the tasks and that the aspectual cues were strong enough to preferentially lead to the expected response (As suggested by an anonymous reviewer due to sentence completion tasks being relatively repetitive, we also ensure that no learning effect was present by comparing participants’ scores on the first part of the task’ items with the last part. The performance of both groups of participants did not become more accurate as they progressed through the tasks. Results from Wilcoxon signed rank tests showed that the differences between the two parts of items are not significant for the four tasks and in both groups (control group: T1 *Z* = − 0.14, *p* > .05; T2 *Z* = − 0.38, *p* > .05; T3 *Z* = − 0.52, *p* > .05; T4 *Z* = − 0.69, *p* > .05; AD group: T1 *Z* = − 0.91, *p* > .05; T2 *Z* = − 1.74, *p* > .05; T3 *Z* = − 1.72, *p* > .05; T4 Z = − 1.29, *p* > .05).).

#### Assessment of cognitive functions

Standardized tests were used to assess cognitive functions to reach our objectives of investigating the contribution of executive functions on time reference. The MoCA^46^ was used to obtain a measure of the general cognitive participant functioning. Verbal short term memory and working memory were assessed with the forward and backward digit span tasks from the Wechsler Memory Scale (WMS-IV)^53^, mental flexibility with the Trail Making Test (version A and B)^54^ and the Category Switching condition of the Verbal Fluency test from the Delis-Kaplan Executive Function System (D-KEFS)^55^, and inhibition with the Stroop Victoria^56^. The order of presentation of these tests was pseudo-randomized between all participants.

To control for a potential effect of visual acuity on task performance, in addition to the inclusion criteria of having normal or corrected vision, a visual gnosis task from the Birmingham Object Recognition Battery^57^ was included in the research protocol for participants who showed visual errors during initial testing. However, based on comprehensive assessments conducted by neuropsychologists and physicians, none of the participants demonstrated visual acuity difficulties to impact their performance on the tasks. Therefore, this additional task was not administered.

### Data analysis

All analyses were performed using R^58^ to address our research objectives.

Non-parametric Wilcoxon rank-sum tests (equivalent to Mann-Whitney U tests) were conducted for two purposes: first, to verify that both groups were matched for age and education level, and second, to compare cognitive performance between the two groups. This approach was chosen following Kolmogorov-Smirnov tests which revealed that the data did not follow a normal distribution. Group (participants with AD and control) was used as the independent variable. Age, education level and scores on tests assessing cognitive functions were used as dependent variables.

To assess performance on verb tense and aspect processing in both groups of participants (AD and controls), we conducted generalized linear mixed models (GLMM) with participant responses to the sentence completion tasks as dichotomic dependent variables (correct response produced “yes/ no” corresponding to 1/0). Random effect for participants was systematically included in the models^59^. Items as random effect and tense or aspect as random slopes were added only if they contributed significantly to the model, according to the likelihood ratio test (LRT) result.

For all tasks, the same procedure was followed. First, we examined cognitive contributions to time reference performance using scores from short-term memory, working memory, mental flexibility, and inhibition tasks as predictors, with significance assessed using LRT. Cognitive scores were centered for the analysis. We also systematically assessed the effect of the interaction between the score at each cognitive task and the group to determine whether a cognitive function had a different effect depending on the group. Given the sample size and potential collinearity between cognitive tasks, we employed a forward stepwise selection procedure. Second, we used LRT to assess main effects of group (AD vs. controls), tense (past, present, future), and aspect (perfective, imperfective), as well as group × tense and group × aspect interactions. Variables were dummy coded with control group, present tense, and perfective aspect as reference categories, respectively.

The *p-*value was considered significant at 0.05. For each outcome, betas, standard errors, odds ratios (OR), 95% confidence intervals, and Nakagawa’s marginal (fixed effects) and conditional (fixed and random effects) R²^60^ are presented in the results section. To note, as a ceiling effect was present in the control group for present tense in Task 2, this level was not considered in the analyses.

For all the analyses assessing the contribution of cognitive performance to time reference skills, only the final models including the significant effects are presented in the results section.

## Results

### Sociodemographic and cognitive function information

Table 3 below shows sociodemographic information of the participants and their performance in the tests assessing cognitive functions. Participants with AD showed significantly lower cognitive performance compared to the control participants.

**Table 3:** Sociodemographic information and performance in cognitive testing in the AD group and the control group.

	Task	AD group (*n* = 21)		Control group (*n* = 21)		Non-parametric tests	
Gender		12 F/9 M		11 F/10 M		NA	NA
		Mean	SD	Mean	SD	W	p-value
Age		71.95	9.09	70.81	8.96	202	0.64
Education		13.48	2.44	13.43	2.42	224	0.93
General cognitive functioning	MoCA^1^	20.33	3.81	28.05	1.56	434.5	**< 0.001**
Short term memory	Digit span forward^2^ (max. = 8)	5.62	0.87	6.24	1.09	302	**< 0.05**
Verbal working memory	Digit span backward^2^ (max. = 8)	3.86	0.73	4.71	1.31	310	**< 0.05**
Flexibility	Category switching fluency D-KEFS^3^	9.05	4.43	16.05	2.97	402.5	**< 0.001**
	TMT (B/A)^4^	2.52	1.35	2.05	0.65	182.5	0.65
Inhibition	Stroop interference score^5^	3.47	1.92	2.15	0.44	153.5	0.12

^1^Nasreddine et al.^46^; ^2^Wechsler^53^; ^3^Delis et al.^55^; ^4^Tombaugh^54^; ^5^Bayard et al.^56^; NA = nonapplicable; SD = standard deviation; W = Wilcoxon rank-sum test; F = female; M = male.

### Tense

**Fig. 3:**
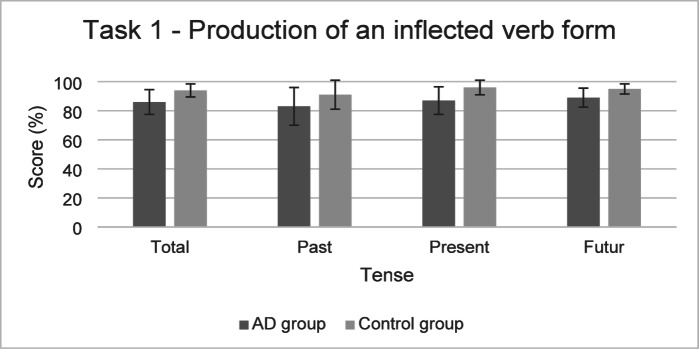
Performance in the Task 1 (mean and standard deviation) in the AD group and the control group.

The performances of both groups for the Task 1 are presented in Fig. 3.

**For Task 1** (Production of an inflected verb form), GLMM statistical models found a significant effect of verbal working memory (*χ*^*2*^(1) = 22.23, *p* < .001) and of mental flexibility assessed with the category switching task (*χ*^*2*^(1) = 10.09, *p* < .01). As reported in Table 4 below, participants with better performance in these tasks also had a higher probability of obtaining a higher score on Task 1. The final model indicated that fixed effects explained 32% of the variance while the inclusion of random effects increased this to 53%.

**Table 4:** Results of the GLMM statistical models (fixed and random effects) for task 1—production of an inflected verb form.

Variables	β	95% CI	SE	Z	OR	*p*-value	*R* ^2^	Random effect variance	Random effect SD
Task 1									
Verbal WM	0.88	[0.74, 1.75]	0.29	3.08	2.41	< 0.01	Conditional: 0.53 Marginal: 0.32	Item: 0.40 Subjects: 0.96	Item: 0.63 Subjects: 0.98
Mental flexibility—category switching task	0.12	[0.08, 0,36]	0.06	1.99	1.13	< 0.05	Conditional: 0.53 Marginal: 0.32	Item: 0.40 Subjects: 0.96	Item: 0.63 Subjects: 0.98

Note. β = intercept; CI = confidence interval; SE = standard error; Z = Z-value; OR = odds ratio; R^2^ = coefficient of determination; SD = standard deviation; WM = working memory.

Likelihood ratio tests also reported insufficient evidence for an effect of the group (*χ*^*2*^(1) = 3.17, *p* = .07), no significant effect of tense (*χ*^*2*^(2) = 3.51, *p* = .17), and of the interaction between the group and tense (*χ*^*2*^(2) = 2.54, *p* = .28).

**Fig. 4:**
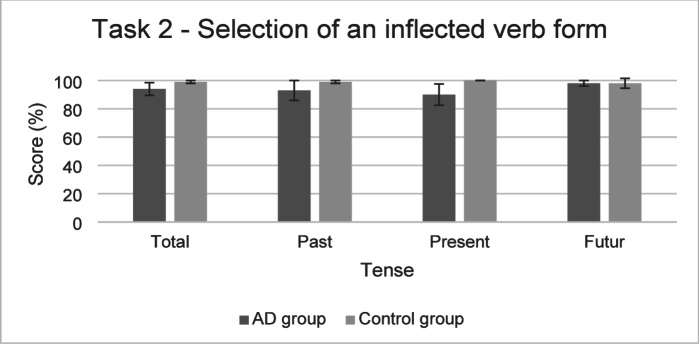
Performance in the Task 2 (mean and standard deviation) in the AD group and the control group.

The performances of both groups for the Task 2 are presented in Fig. 4.

**For Task 2** (Selection of an inflected verb form), the model found a significant effect of flexibility assessed with the TMT (B/A) (*χ*^*2*^(1) = 4.61, *p* < .05). Participants with better performance in this task also had a higher probability of obtaining a higher score on Task 2. For past tense, the model found a significant effect of flexibility assessed with the category switching task (*χ*^*2*^(1) = 5.33, *p* < .05) and a significant effect of the interaction between the group and verbal working memory (*χ*^*2*^(1) = 3.91, *p* < .05). As reported in Table 5 below, participants with better performance in this task also had a higher probability of obtaining a higher score on past tense score on Task 2. The interaction effect between the group and verbal working memory showed that the effect of verbal working memory was only significant in the AD group. The final model indicated that fixed effects explained 27% of the variance while the inclusion of random effects increased this to 56%.

**Table 5:** Results of the GLMM statistical models (fixed and random effects) for task 2—selection of an inflected verb form.

Variables	β	95% CI	SE	Z	OR	*p*-value	*R* ^2^	Random effect variance	Random effect SD
Task 2									
Group	− 1.24	[− 2.45, − 0.04]	0.61	− 2.03	0.29	< 0.001	Conditional: 0.43 Marginal: 0.12	Subjects: 1.71	Subjects: 1.31
Group * Tense	− 2.10	[− 3.96, − 077]	0.89	2.35	8.17	< 0.05	Conditional: 0.43 Marginal: 0.12	Item: 4.61 Subjects: 1.99	Item: 2.15 Subjects: 1.41
Mental flexibility—TMT	− 0.02	[− 0.3, − 0.006]	0.005	− 3.26	0.98	< 0.01	Conditional: 0.56 Marginal: 0.27	Item: 0.09 Subjects: 1.41	Item: 0.31 Subjects: 1.18
Past: Mental flexibility—category switching task	0.23	[0.03, 0.43]	0.10	2.24	1.26	< 0.05	Conditional: 0.56 Marginal: 0.27	Item: 0.40 Subjects: 2.26	Item: 0.63 Subjects: 1.50
Past: group* verbal WM	1.96	[0.34, 3.57]	0.82	2.38	7.12	< 0.05	Conditional: 0.56 Marginal: 0.27	Item: 0.40 Subjects: 2.07	Item: 0.63 Subjects: 1.44

β = intercept; CI = confidence interval; SE = standard error; Z = Z-value; OR = odds ratio; R^2^ = coefficient of determination; SD = standard deviation; WM = working memory.

In this task, likelihood ratio tests also reported a significant effect of the group (*χ*^*2*^(1) = 8.69, *p* < .01) and of the interaction between group and tense (*χ*^*2*^(2) = 9.64, *p* < .01). The significant effect of the group showed that the probability of producing the expected answer was lower in the AD group than in the control group. The interaction effect between the group and tense revealed that the probability of producing the expected answer was only lower in the past condition in the AD group compared to the control group. Insufficient evidence for an effect of tense (*χ*^*2*^(1) = 3.20, *p* = .07) was found. The final model indicated that fixed effects explained 12% of the variance while the inclusion of random effects increased this to 43%.

### Grammatical aspect

**Fig. 5:**
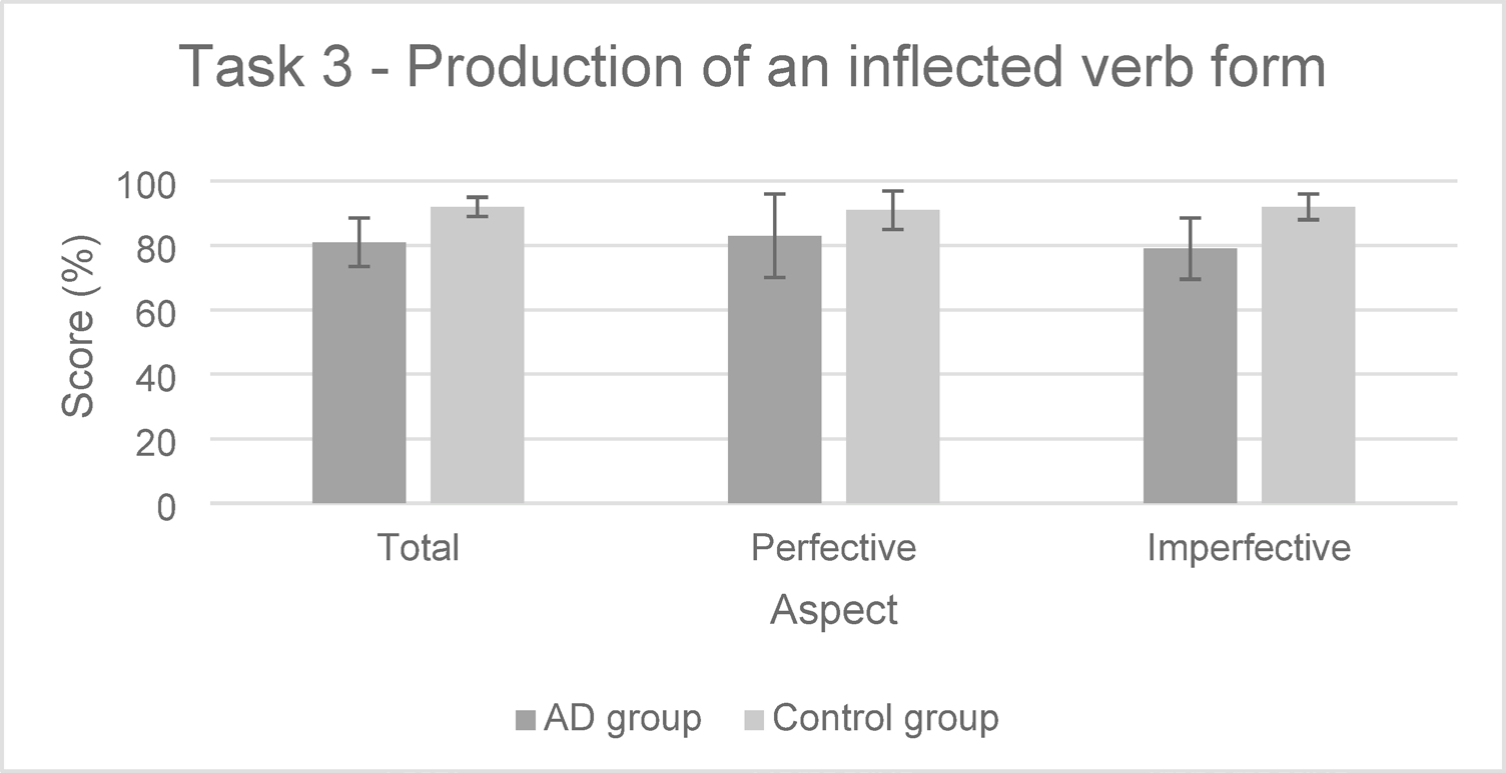
Performance in the Task 3 (mean and standard deviation) in the AD group and the control group.

The performances of both groups for the Task 3 are presented in Fig. 5.

For **Task 3** (Production of an inflected verb form), likelihood ratio tests reported a significant effect of inhibition (*χ*^*2*^(1) = 6.88, *p* < .01). As reported in Table 6, participants having faster time to complete the Stroop task had also higher probability of obtaining a higher score on Task 3. The final model indicated that fixed effects explained 8% of the variance while the inclusion of random effects increased this to 26%.

**Table 6:** Results of the GLMM statistical models (fixed and random effects) for task 3—production of an inflected verb form.

Variables	β	95% CI	SE	Z	OR	*p*-value	*R* ^2^	Random effect variance	Random effect SD
Task 3									
Group	− 0.97	[− 1.83, − 0.42]	0.32	− 0.30	0.37	< 0.01	Conditional: 0.27 Marginal: 0.05	Item: 0.38 Subjects: 0.62	Item: 0.61 Subjects: 0.79
Inhibition - Stroop	− 0.35	[− 0.60, − 0.10]	0.13	− 2.76	0.71	< 0.01	Conditional: 0.26 Marginal: 0.08	Item: 0.46 Subjects: 0.37	Item: 0.68 Subjects: 0.61

β = intercept; CI = confidence interval; SE = standard error; Z = Z-value; OR = odds ratio; R^2^ = coefficient of determination; SD = standard deviation.

GLMM statistical models also showed a significant effect of the group (*χ*^*2*^(1) = 9.09, *p* < .01). The probability of producing the expected answer was lower in the AD group than in the control group. No significant effect of aspect (*χ*^*2*^(1) = 0.01, *p* = .92), and of the interaction between the group and aspect (*χ*^*2*^(2) = 0.82, *p* = .66) was found. The final model indicated that fixed effects explained 5% of the variance while the inclusion of random effects increased this to 27%.

**Fig. 6:**
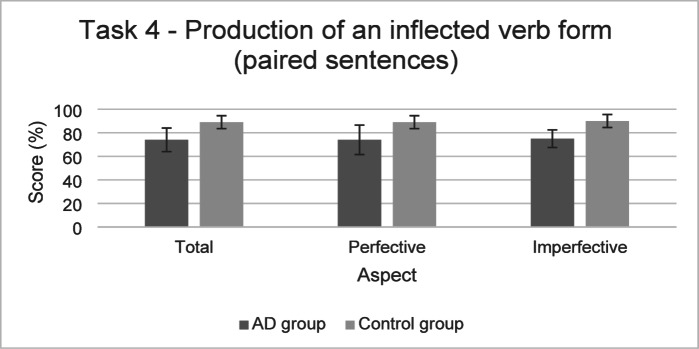
Performance in the Task 4 (mean and standard deviation) in the AD group and the control group.

The performances of both groups for the Task 4 are presented in Fig. 6.

For **Task 4** (Production of an inflected form – aspectual update), likelihood ratio tests reported a significant effect of verbal working memory (*χ*^*2*^(1) = 20.27, *p* < .001). As reported in Table 7, participants with higher performance at this task had also higher probability of obtaining a higher score on Task 4. The final model indicated that fixed effects explained 20% of the variance while the inclusion of random effects increased this to 47%.

**Table 7:** Results of the GLMM statistical models (fixed and random effects) for task 4—production of an inflected verb form (paired sentences).

Variables	β	95% CI	SE	Z	OR	*p*-value	*R* ^2^	Random effect variance	Random effect SD
Task 4									
Group	− 1.35	[− 2.04, − 0.66]	0.35	− 3.83	0.26	< 0.001	Conditional: 0.43 Marginal: 0.08	Item: 1.23 Subjects: 0.85	Item: 1.11 Subjects: 0.92
Verbal WM	0.78	[0.44, 1.12]	0.17	2.77	2.18	< 0.01	Conditional: 0.47 Marginal: 0.20	Item: 1.36 Subjects: 0.31	Item: 1.17 Subjects: 0.56

Note. β = intercept; CI = confidence interval; SE = standard error; Z = Z-value; OR = odds ratio; R^2^ = coefficient of determination; SD = standard deviation; WM = working memory.

GLMM statistical models also found a significant effect of the group (*χ*^*2*^(1) = 12.87, *p* < .001). The probability of producing the expected answer was lower in the AD group than in the control group. No significant effect of aspect (*χ*^*2*^(1) = 0.04, *p* = .84), and of the interaction between the group and aspect (*χ*^*2*^(1) = 0.31, *p* = .58) was found. The final model indicated that fixed effects explained 8% of the variance while the inclusion of random effects increased this to 43%.

## Discussion

This study examined how time reference is processed in AD, with a particular focus on how the cognitive profile of French-speaking individuals with AD affects their ability to produce time reference through tense and grammatical aspect. While previous research has largely examined the linguistic mechanisms underlying time reference – notably in aphasiology, our work shifts attention to the cognitive processes that support these mechanisms in AD. Because expressing time in language requires not only morpho-semantic/syntactic encoding but also complex conceptual and temporal reasoning, time reference is at the interface between language and cognition. Consequently, this study goes beyond merely describing linguistic deficits; it sought, using predictive analyses, to explain why they arise by linking observed linguistic patterns to the underlying cognitive mechanisms such as executive functions. Participants completed four sentence-completion tasks (two for tense, two for grammatical aspect) alongside standardized cognitive assessments. Individuals with AD demonstrated impaired performance across all time reference tasks compared to controls, with executive functions significantly predicting both tense and aspect processing abilities in AD. Interestingly, performances in past tense were more impaired in participants with AD for Task 2 (Selection of an inflected verb form) and predicted by their verbal working memory abilities. These results suggest that time reference impairment in people with AD may be due to executive-function-related deficits rather than to morphosemantic difficulties.

The results of this study demonstrate that producing time reference is a complex cognitive ability that relies on multiple cognitive functions, including verbal working memory, inhibition, and mental flexibility. While previous research has already established the role of working memory in time reference^12,35^, the present findings align with and expand upon these studies. By exploring the influence of additional cognitive functions, this study provides further insight into the processes involved in marking time through language. Beyond the documented role of working memory, our findings highlight the significant contribution of other executive functions, particularly inhibition and mental flexibility. These functions were implicated across all experimental tasks, whether in verb production or selection, suggesting that producing tense and grammatical aspect in French is, at least in part, an executive function-driven process. Fyndanis et al.^12^ previously discussed the possible role of inhibition in verb inflection in AD, particularly in relation to the production of mood (e.g., indicative, subjunctive), but did not investigate it. Although mood was not examined in the present study, inhibition was found to predict performance in verb inflection at aspect level in French. Interestingly, inhibition seems to be particularly involved in Task 3 (Production of an inflected verb form) assessing grammatical aspect, where at least two competing verb forms (perfective and imperfective) exist within the same tense (i.e., past). To produce the correct inflected verb form in this task, the speaker must suppress the inappropriate form that does not match the given aspectual context. This finding aligns with Cortese et al.^41^, who demonstrated that individuals with AD primarily struggle with inhibiting and selecting among competing verb forms, rather than experiencing a fundamental loss of verb knowledge. While inhibition was found to affect verbal inflection, mental flexibility has not been widely discussed in previous studies as a potential factor in this process. However, producing time reference through verbal inflection requires managing a large amount of information and constantly switching between different verbal forms. Our findings suggest that mental flexibility plays a crucial role in time reference, further emphasizing the complexity of time reference production.

In addition, the results indicate that time reference is not fully preserved in French speakers with AD, aligning with previous studies that found an alteration of tense and aspect production in Greek and Italian speakers with AD^12–15^. In the present study, AD participants were able to complete the two tense tasks proposed, but they had greater difficulty than the control group in selecting the correct inflected verb form from three options (Task 2 - Selection of an inflected form). They notably underperformed compared to the control group in the past tense condition in Task 2. Based on the PADILIH^4^, which states that past tense may be a particularly difficult tense to process, and given the executive dysfunction characteristic of AD, we hypothesized that past tenses might be particularly difficult for the AD group. Production of past tenses could indeed demand more processing resources due to the mismatch between the time of the event and the one of utterance of this event. However, our results do not fully support this hypothesis, as no significant difference in tense usage was observed in Task 1 (Production of an inflected verb form). Inflecting a verb in the past tense did not generate significantly more errors than inflecting it in the present or future tense for the participants with AD in our study. The fact remains, that when the amount of information contained in the proposed task increased, past tense became more difficult.

In Task 2, participants were required to analyze the temporal information conveyed by the sentence’s adverbial, as well as that encoded in the three inflected verb forms proposed as possible answers. The observation that a tense effect emerged only in Task 2 is somewhat unexpected, given that production tasks are generally considered more challenging than selection tasks; a view largely supported by findings in aphasiology^6,34,61^. However, the cognitive profiles of people with aphasia and those with AD differ, which may influence how these groups process time reference. Using the same experimental tasks described in the present study, Cordonier and Fossard^6^ found that people with aphasia experienced difficulty producing verbal inflections (Task 1) compared to a control group, while their performance in selecting inflected verb form (Task 2) was less impaired. In this group, a tense effect appeared exclusively in Task 1, with significantly lower performance for the past tense compared to the present and the future tenses. Conversely, in people with AD, the tense effect was observed only in Task 2. These contrasting patterns suggest that verb inflection difficulties in these two populations may stem from different underlying mechanisms. While people with aphasia primarily exhibit morphosemantic difficulties impacting the production of inflected verb forms making Task 1 particularly challenging due to its reliance on active verb inflection generation, participants with AD may face more executive-function-related deficits. They indeed seem to retain relatively intact morphosemantic production abilities, enabling them to correctly perform on Task 1, but have difficulties with Task 2, which requires simultaneous processing of sentence content and multiple response options. The findings regarding working memory performance further support this hypothesis and highlight the complex interplay between task demands and cognitive profiles, even when experimental procedures are consistent across studies. Notably, the two clinical groups exhibit opposite patterns in Task 2: in participants with aphasia, working memory scores did not predict outcomes on this task, whereas they did in participants with AD. Performances of participants with AD in Task 2 may then be due to their compromised working memory resources.

Past tense selection difficulties observed in Task 2 for participants with AD may also be explained by these compromised working memory resources. The effect of the interaction between the group and verbal working memory might support this hypothesis. Indeed, this effect showed that for participants with AD only, verbal working memory ability predicted performance in past tense items in Task 2 (i.e., AD participants with better verbal working memory ability also performed better on the past tense items). These results therefore suggest that past tense processing requires a significant amount of verbal working memory, which places an additional burden on the processing resources needed to perform the task.

Regarding grammatical aspect, participants with AD showed more difficulty than the control group in producing perfective and imperfective verb forms in the past tense (Tasks 3 and 4 - production of an inflected verb form). This suggests that participants had difficulties to produce the inflected verb forms that appropriately matched the aspectual frame conveyed by a temporal adverbial. These findings are consistent with previous studies indicating that grammatical aspect is impaired in Greek speakers with AD^13–15^.

Contrary to expectations, however, the present study did not reveal any differences between the perfective and imperfective aspects, consistent with studies using sentence completion tasks^14,15^. Since referring to an incomplete action might require mentally projecting oneself from the present to the moment of the event - viewing the action from within - we anticipated greater difficulty in producing the imperfective compared to the perfective. The perfective, which denotes a completed action and does not necessitate such projection, was expected to be easier due to lower processing demands. One possible explanation for this finding is that the incomplete sentences used in the proposed tasks offered limited contextual support, thereby minimizing the need for mental projection. In this context, the imperfective, which typically requires more projection because it represents an ongoing action into which one can situate oneself, is not necessarily harder to produce than the perfective, since projection was not a central component of the task (for further discussion on aspect and event representation see for instance^62,63^.

These results suggest that future studies would benefit from examining aspect production in more naturalistic tasks, such as narrative discourse, where richer contextual cues may enhance the opportunity for projection and provide a more sensitive assessment of aspect processing. For instance, employing discursive tasks, such as the Autobiographical Interview^64^ would facilitate a deeper examination of the relationship between time reference and the cognitive cost of projection. While the cognitive demands associated with the projection in verb production can be analyzed from an executive processing perspective, they may also be closely tied to mental time travel. Recounting past or future events requires constructing a mental representation that includes the temporal framework of that event to produce the appropriate verbal forms. This process necessitates disengaging from the present moment and reorienting one’s perspective toward a different temporal context - past or future. Such a shift may influence how events are linguistically encoded, thereby increasing the complexity of verb tense production. Given the cognitive profile associated with AD, particularly the impairment of autobiographical memory and difficulty with mental time travel^65–67^, further research into the link between time reference and mental time travel is warranted^68^.

Several limitations of the present study must be acknowledged. A first limitation concerns the sample size of the clinical group. Despite restrictive inclusion criteria requiring biomarker-confirmed AD and a sample size that is relatively large compared to previous studies, the study remains powered to detect large effects. Accordingly, future research with larger cohorts will be needed to capture more moderate effect sizes and enhance the generalizability of these findings. Secondly, we used sentence completion tasks that constrained participants to produce a specific answer. Although these tasks provide useful information, it would be interesting to use more implicit and ecological tasks. Indeed, the tasks used in the present study required explicit and declarative responses. Such type of tasks were showed to elicit greater age-related deficits and higher cognitive demand compared to more implicit tasks^69,70^. Studying time reference in discursive tasks, in which participants could produce time reference more naturally and implicitly, could help us to better understand the cognitive processes involved. Thirdly, our statistical approach presents important limitations. We employed stepwise selection procedures to identify cognitive processes and their interaction effects associated with verb inflection abilities, as including all predictors simultaneously resulted in model convergence failures due to sample size constraints relative to model complexity. While this approach allowed us to handle our data structure, stepwise procedures are associated with increased risk of Type I errors, potential overfitting, and instability of selected models^71^. The significant effects reported here should therefore be interpreted cautiously, and replication in larger samples would strengthen confidence in these findings. Finally, this study focused on the implication of executive functions on time reference in AD. Other cognitive functions may also be implicated in time reference. Indeed, as telling past or future events requires to build mental representations including temporal and personal information, semantic and episodic memory, which are linked to autobiographical memory may also play a part in the production of time reference. It would therefore be relevant to use tasks combining a natural production of time reference and the implication of autobiographical memory, such as the Autobiographical Interview^64^.

## Conclusion

In conclusion, this study demonstrates that producing time reference is a cognitive complex process. More specifically, it shows that French speakers with AD have some difficulty with tense and aspect marking and that verbal working memory, inhibition, and mental flexibility may play a crucial role in time reference. Further research is needed to clarify how the cognitive profiles of individuals with AD influence their ability to express time reference and to explore the potential relationship between mental time travel and time reference processing in AD.

## Data Availability

The data and material supporting the findings of this study are available on request from the principal investigator, Prof. Marion Fossard, on SWISSUbase (Project 20962)^72,73^. The data are not publicly available due to privacy or ethical restrictions.

## References

[1] Conway, M. A. Memory and the self. *J. Mem. Lang.* **53** (4), 594–628 (2005).

[2] Prebble, S. C., Addis, D. R. & Tippett, L. J. Autobiographical memory and sense of self. *Psychol. Bull.* **139** (4), 815–840 (2013). 23025923 10.1037/a0030146

[3] Auclair-Ouellet, N., Pythoud, P., Koenig-Bruhin, M. & Fossard, M. Inflectional morphology in fluent aphasia: A case study in a highly inflected Language. *Lang. Speech*. **62(2)** (1), 250–259 (2019). 10.1177/0023830918765897 29577804

[4] Bastiaanse, R. et al. Time reference in agrammatic aphasia: A cross-linguistic study. *J. Neurolinguistics*. **24** (6), 652–673 (2011). 26451073 10.1016/j.jneuroling.2011.07.001 PMC4594877

[5] Cordonier, N., Ericson, C., Schneider, L., Bellmann, A. & Fossard, M. Time reference in French-speaking people with fluent and non-fluent aphasia (part II): a cluster analysis. *Aphasiology* **39** (8), 1097–1121 (2024).

[6] Cordonier, N. & Fossard, M. Time reference in French-speaking people with fluent and non-fluent aphasia (part I): tense dissociations, task effects and cognitive predictors. *Aphasiology* **39** (8), 1066–1096 (2024).

[7] Faroqi-Shah, Y. & Thompson, C. K. Semantic, lexical, and phonological influences on the production of verb inflections in agrammatic aphasia. *Brain Lang.* **89** (3), 484–498 (2004). 10.1016/j.bandl.2003.12.006 PMC3025186 15120539

[8] Faroqi-Shah, Y. & Thompson, C. K. Verb inflections in agrammatic aphasia: encoding of tense features. *J. Mem. Lang.* **56** (1), 129–151 (2007). 18392120 10.1016/j.jml.2006.09.005 PMC2288584

[9] Fyndanis, V. et al. Time reference in nonfluent and fluent aphasia: a cross-linguistic test of the PAst DIscourse LInking Hypothesis. Clin Linguist Phon. 2018 Sept 2;32(9):823–43. 10.1080/02699206.2018.1445291 29513613

[10] Auclair-Ouellet, N. Inflectional morphology in primary progressive aphasia and alzheimer’s disease: A systematic review. *J. Neurolinguistics*. **34**, 41–64 (2015).

[11] Auclair-Ouellet, N., Macoir, J., Laforce, R., Bier, N. & Fossard, M. Regularity and beyond: impaired production and comprehension of inflectional morphology in semantic dementia. *Brain Lang.* **155–156**, 1–11 (2016). 26994740 10.1016/j.bandl.2016.02.002

[12] Fyndanis, V. et al. Morphosyntactic production in Greek- and Italian-speaking individuals with probable alzheimer’s disease: evidence from subject–verb agreement, tense/time reference, and mood. *Aphasiology* **32** (1), 61–87 (2018).

[13] Fyndanis, V., Manouilidou, C., Koufou, E., Karampekios, S. & Tsapakis, E. M. Agrammatic patterns in alzheimer’s disease: evidence from tense, agreement, and aspect. *Aphasiology* **27** (2), 178–200 (2013).

[14] Manouilidou, C., Roumpea, G., Nousia, A., Stavrakaki, S. & Nasios, G. Revisiting aspect in mild cognitive impairment and alzheimer’s disease: evidence from Greek. *Front. Commun.* **5**, 434106 (2020).

[15] Roumpea, G., Nousia, A., Stavrakaki, S., Nasios, G. & Manouilidou, C. Lexical and grammatical aspect in mild cognitive impairment and alzheimer’s disease. *Sel. Pap Theor. Appl. Linguist* **23**, 381–397 (2019).

[16] McKhann, G. M. et al. The diagnosis of dementia due to alzheimer’s disease: recommendations from the National Institute on Aging-Alzheimer’s association workgroups on diagnostic guidelines for alzheimer’s disease. *Alzheimers Dement.* **7** (3), 263–269 (2011). 21514250 10.1016/j.jalz.2011.03.005 PMC3312024

[17] Grober, E. et al. Memory impairment, executive dysfunction, and intellectual decline in preclinical Alzheimer’s disease. *J. Int. Neuropsychol. Soc.* **14**(2), 266–78. (2008). 10.1017/S1355617708080302 PMC2763488 18282324

[18] Wilson, R. S., Leurgans, S. E., Boyle, P. A. & Bennett, D. A. Cognitive decline in prodromal alzheimer disease and mild cognitive impairment. *Arch. Neurol.* **68** (3), 351–356 (2011). 21403020 10.1001/archneurol.2011.31 PMC3100533

[19] Almor, A., Kempler, D., MacDonald, M. C., Andersen, E. S. & Tyler, L. K. Why do alzheimer patients have difficulty with pronouns? Working Memory, Semantics, and reference in comprehension and production in alzheimer’s disease. *Brain Lang.* **67** (3), 202–227 (1999). 10210631 10.1006/brln.1999.2055

[20] MacDonald, M. C., Almor, A., Henderson, V. W., Kempler, D. & Andersen, E. S. Assessing working memory and Language comprehension in alzheimer’s disease. *Brain Lang.* **78** (1), 17–42 (2001). 10.1006/brln.2000.2436 11412013

[21] Van Boxtel, W. & Lawyer, L. Sentence comprehension in ageing and Alzheimer’s disease. *Lang Linguist Compass [Internet].* **15**(6). 10.1111/lnc3.12430 (2021).

[22] Comrie, B. & Aspect *An Introduction To the Study of Verbal Aspect and Related Problems* (Cambridge University Press, 1976). (Cambridge Textbooks in Linguistics).

[23] Grisot, C. & Cohesion Coherence and Temporal Reference from an Experimental Corpus Pragmatics Perspective [Internet]. Cham: Springer International Publishing; [cited 2023 July 4]. (Yearbook of Corpus Linguistics and Pragmatics). Available from: 10.1007/978-3-319-96752-3 (2018).

[24] Vetters, C. & Temps aspect et narration. Amsterdam-Atlanta: Rodopi; (Faux titre: etudes de langue et littérature françaises publiées). (1996).

[25] Dragoy, O. & Bastiaanse, R. Aspects of time: time reference and aspect production in Russian aphasic speakers. *J. Neurolinguistics*. **26** (1), 113–128 (2013).

[26] Fyndanis, V. & Themistocleous, C. Are there prototypical associations between time frames and aspectual values? Evidence from Greek aphasia and healthy ageing. *Clin. Linguist Phon*. **33** (1–2), 191–217 (2019). 29939796 10.1080/02699206.2018.1480657

[27] Klein, W. Time in Language, Language in time. *Lang. Learn.* **58**, 1–12 (2008).

[28] Reichenbach, H. *Elements of Symbolic Logic* (Mcmillan Compagny, 1947).

[29] Taler, V. & Phillips, N. A. Language performance in alzheimer’s disease and mild cognitive impairment: A comparative review. *J. Clin. Exp. Neuropsychol.* **30**(5), 501–556 (2008). 10.1080/13803390701550128 18569251

[30] Ahmed, S., Haigh, A. M. F., De Jager, C. A. & Garrard, P. Connected speech as a marker of disease progression in autopsy-proven alzheimer’s disease. *Brain* **136** (12), 3727–3737 (2013). 24142144 10.1093/brain/awt269 PMC3859216

[31] Sajjadi, S. A., Patterson, K., Tomek, M. & Nestor, P. J. Abnormalities of connected speech in semantic dementia vs Alzheimer’s disease. *Aphasiology.* **26**(6), 847–66 (2012).

[32] Ullman, M. T. et al. A neural dissociation within language: evidence that the mental dictionary is part of declarative Memory, and that grammatical rules are processed by the procedural system. *J. Cogn. Neurosci.* **9** (2), 266–276 (1997). 23962016 10.1162/jocn.1997.9.2.266

[33] Pinker, S. Words and rules. *Lang. Acquis Knowl. Represent Process.* **106** (1), 219–242 (1998).

[34] Cordonier, N., Schaffner, E., Zeroual, L. & Fossard, M. Time reference in aphasia: are there differences between tenses and aphasia fluency type? A systematic review and individual participant data meta-analysis. *Front. Psychol.* **15**. (2024). 10.3389/fpsyg.2024.1322539 PMC10885357 38406299

[35] Fyndanis, V., Arcara, G., Christidou, P. & Caplan, D. Morphosyntactic production and verbal working memory: evidence from Greek aphasia and healthy aging. *J. Speech Lang. Hear. Res.* **61** (5), 1171–1187 (2018). 29710332 10.1044/2018_JSLHR-L-17-0103

[36] Kok, P., Vandoorn, A. & Kolk, H. Inflection and computational load in agrammatic speech. *Brain Lang.* **102** (3), 273–283 (2007). 10.1016/j.bandl.2007.03.001 17433430

[37] Fyndanis, V., Varlokosta, S. & Tsapkini, K. Agrammatic production: interpretable features and selective impairment in verb inflection. *Lingua* **122** (10), 1134–1147 (2012).

[38] Avrutin, S. Comprehension of discourse-linked and non-discourse-linked questions by children and broca’s aphasics. In: (eds Grodzinsky, Y., Shapiro, L. P. & Swinney, D.) Language and the Brain: Representation and Processing. Academic. San Diego; 295–313. (2000).

[39] Avrutin, S. Weak syntax. In: (eds Amunts, K. & Grodzinsky, Y.) Broca’s Region. Oxford. New York; 49–62. (2006).

[40] Coelho, S. et al. Time perspective and amnestic mild cognitive impairment. *J. Neuropsychol.* **16**(3), 463–480 (2022). 10.1111/jnp.12274 35174621

[41] Cortese, M. J., Balota, D. A., Sergent-Marshall, S. D., Buckner, R. L. & Gold, B. T. Consistency and regularity in past-tense verb generation in healthy ageing, alzheimer’s disease, and semantic dementia. *Cogn. Neuropsychol.* **23** (6), 856–876 (2006). 10.1080/02643290500483124 21049357

[42] El Haj, M., Antoine, P. & Kapogiannis, D. Flexibility decline contributes to similarity of past and future thinking in alzheimer’s disease. *Hippocampus* **25** (11), 1447–1455 (2015). 25850800 10.1002/hipo.22465 PMC5460916

[43] Irish, M. et al. Language of the past’ - Exploring past tense disruption during autobiographical narration in neurodegenerative disorders. *J. Neuropsychol.* **10** (2), 295–316 (2016). 10.1111/jnp.12073 26014271

[44] American Psychiatric Association. Diagnostic and statistical manual of mental disorders. 5th edition. (2013).

[45] American Psychiatric Association. Diagnostic and statistical manual of mental disorders. Text revision. 5th edition. (2022).

[46] Nasreddine, Z. S. et al. The Montreal cognitive Assessment, moca: a brief screening tool for mild cognitive impairment. *J. Am. Geriatr. Soc.* **53** (4), 695–699 (2005). 15817019 10.1111/j.1532-5415.2005.53221.x

[47] Jack, C. R. et al. NIA-AA research framework: toward a biological definition of alzheimer’s disease. *Alzheimers Dement.* **14** (4), 535–562 (2018). 29653606 10.1016/j.jalz.2018.02.018 PMC5958625

[48] Macoir, J. et al. Detection test for Language impairments in adults and the Aged—A new screening test for Language impairment associated with neurodegenerative diseases: validation and normative data. *Am. J. Alzheimers Dis. Other Demen*. **32** (7), 382–392 (2017). 28639484 10.1177/1533317517715905 PMC10852687

[49] Macoir, J. et al. Normative data for healthy French-Speaking persons aged 80 years and older for the DTLA Language screening test. *Arch. Clin. Neuropsychol.* **37** (7), 1601–1607 (2022). 35652614 10.1093/arclin/acac036

[50] Schaffner, E. et al. Tense Production in French-speaking Participants with Alzheimer’s Disease: What about discourse? Contribution of a storytelling-in-sequence Task. J Alzheimers Dis. accepted. 10.1177/13872877251376028 PMC12647377 40924821

[51] New, B., Pallier, C., Brysbaert, M. & Ferrand, L. Lexique 2: A new French lexical database. *Behav. Res. Methods Instrum. Comput.* **36** (3), 516–524 (2004). 15641440 10.3758/bf03195598

[52] Schaffner, E., Reber, A., Sandoz, M., Cordonier, N. & Fossard, M. Compléter le puzzle de l’évaluation logopédique dans les troubles acquis du langage - Validation d’une batterie d’évaluation du marquage grammatical de la référence temporelle en français. Poster presentation presented at: 10ème Ecole internationale d’été en logopédie/orthophonie; July 2; Neuchâtel. (2025).

[53] Wechsler, D. Wechsler adul intelligence Scale - Fourth edition (WAIS-IV). *APA PsycTests* (2008).

[54] Tombaugh, T. N. & Trail Making Test, A. Normative data stratified by age and education. *Arch. Clin. Neuropsychol.* **19** (2), 203–214 (2004). 15010086 10.1016/S0887-6177(03)00039-8

[55] Delis, D. C., Kaplan, E. & Kramer, J. H. *The Delis-Kaplan Executive Function System* (Psychological Corporation, 2001).

[56] Bayard, S., Erkes, J., Moroni, C. & the College des Psychologues Cliniciens specialises en Neuropsychologie du Languedoc Roussillon (CPCN Languedoc Roussillon). Victoria Stroop test: normative data in a sample group of older people and the study of their clinical applications in the assessment of Inhibition in alzheimer’s disease. *Arch. Clin. Neuropsychol.* **26**(7), 653–661 (2011). 21873625 10.1093/arclin/acr053

[57] Humphreys, G. & Riddoch, J. M. Birmingham Object Recognition Battery (BORB) [Internet]. APA PsycTests. (1993). Available from: 10.1037/t13731-000.

[58] R Core Team. R: A language and Environment for Statistical Computing [Internet]. Vienna, Austria: R Foundation for Statistical Computing. Available from: https://www.R-project.org/ (2023).

[59] Baayen, R. H., Davidson, D. J. & Bates, D. M. Mixed-effects modeling with crossed random effects for subjects and items. *Spec. Issue Emerg. Data Anal.* **59** (4), 390–412 (2008).

[60] Nakagawa, S. & Schielzeth, H. A general and simple method for obtaining *R*^2^ from generalized linear mixed-effects models. O’Hara RB, editor. *Methods Ecol Evol.* **4**(2), 133–142 (2013).

[61] Cho-Reyes, S. & Thompson, C. K. Verb and sentence production and comprehension in aphasia: Northwestern assessment of verbs and sentences (NAVS). *Aphasiology* **26** (10), 1250–1277 (2012). 26379358 10.1080/02687038.2012.693584 PMC4569132

[62] Magliano, J. P. & Schleich, M. C. Verb aspect and situation models. *Discourse Process.* **29** (2), 83–112 (2000).

[63] Madden, C. J. & Zwaan, R. A. How does verb aspect constrain event representations? *Mem Cognit.* **31**(5), 663–672 (2003). 10.3758/bf03196106 12956232

[64] Levine, B., Svoboda, E., Hay, J. F., Winocur, G. & Moscovitch, M. Aging and autobiographical memory: dissociating episodic from semantic retrieval. *Psychol. Aging*. **17** (4), 677–689 (2002). 12507363

[65] Addis, D. R., Sacchetti, D. C., Ally, B. A., Budson, A. E. & Schacter, D. L. Episodic simulation of future events is impaired in mild alzheimer’s disease. *Neuropsychologia* **47** (12), 2660–2671 (2009). 19497331 10.1016/j.neuropsychologia.2009.05.018 PMC2734895

[66] El Haj, M., Antoine, P., Nandrino, J. L. & Kapogiannis, D. Autobiographical memory decline in alzheimer’s disease, a theoretical and clinical overview. *Ageing Res. Rev. ***23**, 183–192 (2015). 10.1016/j.arr.2015.07.001 PMC5456120 26169474

[67] Irish, M. et al. Evolution of autobiographical memory impairments in alzheimer’s disease and frontotemporal dementia – A longitudinal neuroimaging study. *Spec. Issue Autobiographical Mem.* **110**, 14–25 (2018). 10.1016/j.neuropsychologia.2017.03.014 28288787

[68] Schaffner, E., Sandoz, M., Grisot, C., Auclair-Ouellet, N. & Fossard, M. Mental time travel and time reference difficulties in alzheimer’s disease: are they related? A systematic review. *Front. Psychol.* **13**, 858001 (2022). 35615204 10.3389/fpsyg.2022.858001 PMC9126194

[69] Ward, E. V., Berry, C. J. & Shanks, D. R. Age effects on explicit and implicit memory. *Front. Psychol. [Internet].*10.3389/fpsyg.2013.00639/abstract (2013). 10.3389/fpsyg.2013.00639 PMC3779811 24065942

[70] Van Boxtel, W. S. & Lawyer, L. A. A Matter of Memory? Age-Invariant Relative Clause Disambiguation and Memory Interference in Older Adults. *Top. Cogn. Sci.*. 10.1111/tops.12753 (2024).10.1111/tops.1275339415427

[71] Whittingham, M. J., Stephens, P. A., Bradbury, R. B. & Freckleton, R. P. Why do we still use Stepwise modelling in ecology and behaviour? *J. Anim. Ecol.* **75** (5), 1182–1189 (2006). 10.1111/j.1365-2656.2006.01141.x 16922854

[72] Fossard, M., Schaffner, E., Sandoz, M. & Cordonier, N. Constraint tasks [Internet]. UNINE data service; 2025 [cited 2025 Feb 13]. Available from: https://www.swissubase.ch/en/catalogue/studies/20962/latest/datasets/2793/3570/overview.

[73] Fossard, M., Schaffner, E., Sandoz, M. & Cordonier, N. Language and cognitive tests [Internet]. UNINE data service; 2025 [cited 2025 Feb 13]. Available from: https://www.swissubase.ch/en/catalogue/studies/20962/latest/datasets/2797/3560/overview.

